# Change in mineral composition and cooking quality in legumes grown on semi-arid alfisols due to elevated CO_2_ and temperature

**DOI:** 10.3389/fnut.2024.1444962

**Published:** 2025-01-06

**Authors:** K. Sreedevi Shankar, M. Vanaja, Mekala Shankar, Asma Siddiqua, K. L. Sharma, V. Girijaveni, V. K. Singh

**Affiliations:** ^1^ICAR-Central Research Institute for Dryland Agriculture, Hyderabad, Telangana, India; ^2^Jaya Shankar Telangana State Agricultural University, Hyderabad, Telangana, India

**Keywords:** elevated CO_2_, elevated temperature, free air temperature elevation, nutritional quality, principal component analysis

## Abstract

This study aimed to determine the effects of elevated carbon dioxide (eCO_2_) and temperature (eT) on the phytochemical and nutritional parameters of legumes. Field experiments were conducted using black gram (*Vigna mungo* L.), green gram (*Vigna radiate* L.), and pigeonpea (*Cajanus cajan* L.) genotypes under the Free Air Temperature Elevation (FATE) facility, with three treatments (Ac, eT, and eCO_2_ + eT) at ICAR-CRIDA, Hyderabad. The results revealed that the negative impact on both phytochemical and nutritional quality was greater under eT compared to eCO_2_ + eT. Specifically, protein content decreased by 25.6% under eT + eCO_2_, while the ash content increased by 38.19%. Carbohydrate levels also decreased by 5.53% under these conditions_._ The reduction in micronutrients (Zn, Fe, Mn, and Cu) was more pronounced than in macronutrients (P, Ca, and Mg) across the three crops. Moreover, principal component analysis (PCA) revealed that the major contributors to PC1 were Mg, crude fiber, cooking time, phosphorus, hydration capacity, ash content, and Mn. The primary contributors to PC2 included swelling capacity, Cu, Mn, carbohydrate, hydration capacity, and Zn. In contrast, the major contributors to PC3 were Ca, Fe, Zn, protein, carbohydrate, swelling index, and ash content. The eigenvalues of principal components, calibrated through different parameters, ranged from 1.052 to 4.755 in black gram and from 1.073 to 6.267 in green gram. This study provides insights into nutritional quality under changing global climate conditions.

## 1 Introduction

Global warming, with rising levels of carbon dioxide and temperatures, affects crop yield, yet its impact on nutritional quality remains unclear and requires further research. Increased atmospheric CO_2_ concentration and elevated air/soil temperatures directly or indirectly affect the ecosystem (2). Future climatic conditions may adversely affect plant growth and development, human comfort, and ecosystem functions (3). Since the Industrial Revolution, the global annual mean concentration of carbon dioxide has increased significantly from 270 μmol mol^−1^, with global emissions reaching 35.8 Gt CO_2_ per/year (4).

Global climate change models predict that atmospheric CO_2_ levels could reach 700 ppm by 2,100, with temperatures rising by 2.6° to 4.8°C by 2065 and the end of the 21^st^ century, respectively (38). These changes pose a significant threat to water resource availability, plant growth, and fecundity worldwide (5), thereby endangering both food security and nutritional security.

Changes in ambient CO_2_ concentrations directly impact plant physiology and growth, as CO_2_ is a key reactant in photosynthesis (6). Soba et al. (7) revealed that exposing plants to short-term elevated CO_2_ (eCO_2_) levels (700 μmol mol^−1^) significantly increased aboveground biomass and grain yield components while only modestly influencing the biochemical composition of mature grain.

An increased atmospheric carbon dioxide concentration (eCO_2_) would stimulate crop growth through carbon fertilization (8). The adverse effect of the associated global warming due to elevated CO_2_ is also of concern. It offsets the positive impact of eCO_2_ in terms of excessive heat and drought. Thus, studying the crop responses to the eCO_2_ and eT is important.

In a study, soybean grown under elevated [CO_2_] conditions and subjected to high- (+9°C) and low (+5°C) intensity heat waves during key temperature-sensitive reproductive stages (R1, flowering; R5, pod-filling) showed reduced yields under high-intensity heat waves compared to ambient conditions, even with eCO_2_. This reduction was primarily due to heat stress affecting reproductive processes, especially during the R5 stage.

Moreover, low-intensity heat waves applied during R5 uncoupled the negative effects of heating on cellular- and leaf-level processes from plant-level carbon assimilation (9). Jiang et al. (40) revealed that elevated temperatures partially offset the beneficial eCO_2_ effects in most cases. In addition to the direct impact of the eCO_2_ on the global temperature, the CO_2_ plays a unique role in the growth of plants. This is because it would rather play an important role in photosynthesis, producing the sugars, complex carbohydrates, and carbon skeletons for most of the organic compounds in the plants. Studies have focused on understanding the CO_2_ effects on various aspects of plant growth, apart from the productivity and survival of different crops. Few studies have shown that eCO_2_ and eT will have interactive effects on crops that are mostly negative (41, 42).

Moreover, eCO_2_ and eT due to global climate change are expected to significantly affect crop yields and grain quality (10). The individual and interactive effects of elevated CO_2_ (800 ppm), drought (50% field capacity), and heat (40°C) on two C3 (rice and green gram) and two C4 crops (maize and ragi) were studied, and it was found that drought + elevated CO_2_ caused a sharp decline in photosynthetic rate and stomatal conductance in C4 crops, while C3 crops were not similarly affected. However, pollen viability and pollen tube germination were negatively impacted under the combined effects of heat, drought, and elevated CO_2_ levels, which also led to decreased yield traits in both C3 (rice and green gram) and C4 (maize and ragi) crop species (43). Thus, studying the effects of both eCO_2_ and eT is essential to better understand crop responses and ensure crop productivity.

The Free Air CO_2_ Enrichment (FACE) facility is the best approach to assessing the actual response of crops to climate change as it simulates future high CO_2_ environments and temperatures in an open field and is considered the best approach to assessing the actual response of crop production to climate change. Moreover, the Free Air Temperature Elevation (FATE) facility can be a more realistic approach to studying the impact of eT and eCO_2_ (11) as it does not alter microclimate. FACE experiments are costly; thus, there are few studies on diverse regions and conditions. Even conflicting results on nutritional shifts from FACE experiments suggest in-depth research to understand the mechanisms and environmental conditions that result in lower nutrient content in elevated CO_2_/temperature conditions (11). A meta-analysis of 20 years of FACE studies revealed that eCO_2_ increased yield by 16.7% at ambient temperature but only 10.1% at eT (1–2°C) in the case of rice (12). It is thus obvious that the eCO_2_ and eT would have a significant influence on the biochemical composition of grains and their nutritional quality. Elevated CO_2_ levels reduced chlorophyll, magnesium, and phosphorus concentrations, reducing nitrogen concentration (by approximately 39.7%) in sweet potatoes and increasing tuber yield by 20.3% (13). A growing body of evidence suggests that eCO_2_ and high temperatures influence food composition (14). It has been hypothesized that the expected increase of eCO_2_ and temperature in upcoming years might affect several crops' yield, nutritional quality, and contents of carbohydrates, protein, and lipids (39). Climate change positively affects grain legumes. Being C3 crops, they can allocate above-ground photosynthates to below-ground parts, such as roots and nodules, leading to improved plant biomass rhizospheric activities. Nevertheless, the impact of eT on grain legumes is not favorable. High temperatures, especially during the reproductive phase, negatively affect grain legume quality and performance (15).

However, literature containing evidence of the combined effects of both eCO_2_ and high temperature on grain yield and nutritional composition is limited.

A clear understanding of how crop species would respond to global environmental changes would be crucial to developing or modifying the genetic makeup of crops to attain sustainable yields without any reduction in nutritional quality under eCO_2_ and eT. Chaturvedi et al. (16) studied the interaction effect of eCO_2_ and heat stress (HT) on rice in an open-top chamber (OTC) and found that the proportion of chalky grains was further increased under eCO_2_ + HT with a negative effect on grain and nutrient quality in rice. To date, very little information is available on the impact of eCO_2_ and eT in legumes. Legumes are considered one of the world's most important food supplies in developing nations. Legumes are protein-rich food crops that improve the human diet, and their cultivation greatly benefits soil health through their unique ability to fix nitrogen in the soil. In addition, several legume crops are highly resilient to adverse climatic conditions, such as different levels of drought, and crops grown in different dry regions worldwide. Growing legumes is a cost-effective option for improving the diets of low-income consumers who cannot easily afford protein sources such as meat, dairy products, and fish. Legumes are rich in protein and micronutrients such as iron and zinc. They also supply amino acids deficient in cereals, sharply improving the overall protein quality when consumed together. A high content of iron and zinc is highly beneficial for both women and children to avoid the risk of anemia. Legumes also contain the most desired bioactive compounds, which help combat cancer, diabetes, and heart diseases. Moreover, the information related to eCO_2_ and eT under FACE or controlled environments is mostly restricted to phenological and yield parameters but not nutritional quality. Thus, we attempted to study the effect of both eCO_2_ and eT on nutritional quality in three legumes such as green gram, black gram, and pigeon pea.

## 2 Materials and methods

### 2.1 Experimental details

**Figure 1:**
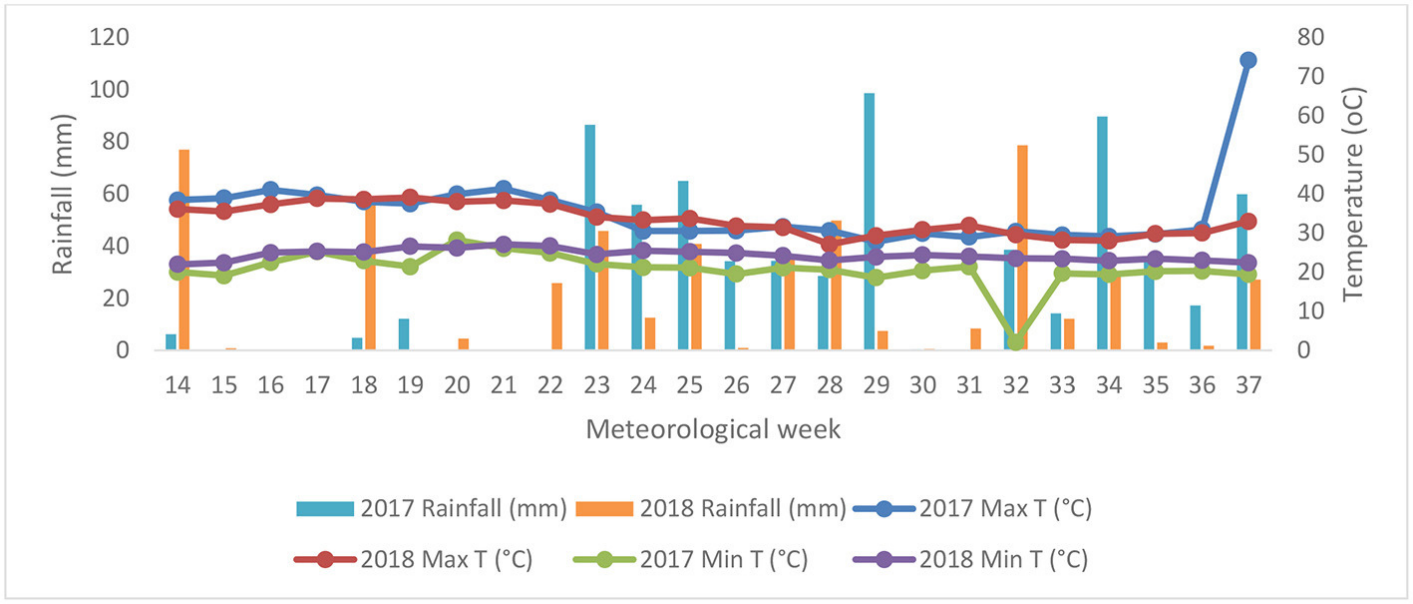
Weekly average minimum temperature, maximum temperature, and rainfall during the crop cycle in 2017 and 2018.

Field experiments were conducted over a course of 2 years in 2017 and 2018. The experiment was in the Hayathnagar Research Farm (17.20°N latitude and 78.3°E longitude) of the Central Research Institute for Dryland Agriculture (CRIDA), Hyderabad, India. For this study, the Free Air Temperature Elevation (FATE) chamber facility was used to assess the impact of elevated crop canopy temperature (eT) and its interaction with the eCO_2_ (eT + eCO_2_). The treatments include control (ambient conditions), eT, and eT + eCO_2_. Their effect on phytochemical and nutritional parameters on three pulse crops, such as black gram (*Vigna mungo* L.), green gram (*Vigna radiate* L.), and pigeon pea (*Cajanus cajan* L.), was studied. Two genotypes, each of black gram (LBG−752 and T−9) and green gram (LGG−460 and WGG−42), and one genotype of pigeonpea (PRG-176) were tested in the study. The experimental design was randomized block design (RBD) with three replications. A maximum temperature of 35.6^0^C was observed at the initial growth stage during the crop growing period. The canopy temperature of eT was elevated by 3^0^C ± 0.5^0^C in the 8 m FATE rings fitted with an array of 24 infrared (IR) heaters, while the CO_2_ was maintained at 550 μmol ${\text{mol}}_{.}^{-\text{1}}$ A reference plot with similar fittings without warming has served as an ambient control (aT). The meteorological data during the crop growth period is given in Figure 1. Experimental soils were sandy loam in texture, neutral in reaction (pH 7.5), non-saline (electrical conductivity 0.20 dS m^−1^), low in organic C (4.1 g kg^−1^), low in available N (156 kg ha^−1^), high in available P (54 kg ha^−1^), high in available K (216 kg ha^−1^) and sufficient in available S (12 mg kg^−1^), available Zn (0.55 mg kg^−1^), and available B (1.4 mg kg^−1^). Post-harvest grain samples were analyzed for mineral content and cooking quality during the study period.

### 2.2 Estimation of the minerals

The metallic cations were determined in the di-acid digest of the grain sample using atomic absorption spectroscopy (1). A dried and processed legume grain sample of 0.5 g was weighed in a 100 ml conical flask. To this, 10 ml of concentrated HNO_3_ was added and placed in a funnel on the conical flask. The samples were kept on a hot plate in the acid-proof digestion chamber, having a fume exhaust system, and heated at 100°C for the 1^st^ h and then rose to 200°C. Appropriate care was taken so that the samples did not dry. The samples were removed from the hot plate, cooled, and filtered through the Whatman No. 42 filter paper into a 100-ml volumetric flask. The digested samples were given 3–4 washings of 15–20 ml portions of the distilled water, and the volume was made up to a level of 100 ml. The readings of plant samples were taken using atomic absorption spectroscopy (model: Perkin Elmer, Analyst 800).

### 2.3 Estimation of phosphorus

Phosphorus in the grain samples was determined using the Vanado-Molybdo Phosphoric Yellow Color method (37) with a spectrophotometer at 420 nm. A 30-ml aliquot was transferred to a 50-ml volumetric flask, to which 10 ml of the vanadate-molybdate solution was added and then diluted to 50 ml with water. The solution was mixed thoroughly, and the color intensity was measured after 10 min using a blue filter at 420 nm. A blank sample was run simultaneously without the phosphorus solution.

### 2.4 Estimation of protein

Nitrogen was estimated using the Kjeldahl method, while the crude protein was calculated based on the formula (N × 6.25) (AOAC, 2001). A 0.5 gm of the legume grain sample was accurately weighed and placed in a clean and dry Kjeldahl flask. Approximately 25 ml of the concentrated H_2_SO_4_ and a few crystals of CuSO4 were added. The flask was heated slowly for a few minutes and heated strongly on open wire gauze for 4 h, and it was subsequently allowed to cool. The flask's content was diluted to 250 ml with distilled water in a volumetric flask. A blank solution was titrated against 0.1 N H_2_SO_4_. The crude protein content has been calculated with a conversion factor of N × 6.25.

### 2.5 Estimation of crude fiber

The crude fiber was estimated using the ANKOM system (17). It was calculated as a loss of the weight of organic matter (W3).


$$\begin{array}{l} \text{The percent crude fiber }=\;100\text{ x }\left({\text{W}}_{3}\;-\;\left({\text{W}}_{1}\text{ x }{\text{C}}_{1}\right)\right)/{\text{W}}_{2}. \end{array}$$

Here, W_1_= bag tare weight, W_2_ = sample weight, W_3_ = weight of organic matter (loss of weight on the ignition of bag and fiber); C_1_ = ash corrected blank bag factor (running average of loss of weight on ignition of blank bag/original blank bag).

### 2.6 Estimation of carbohydrates

The total carbohydrates have been estimated using the phenol sulfuric acid (61). The 100 mg sample was placed into a boiling tube and hydrolyzed in a boiling water bath for 3 h with 5 ml of 2.5 N HCl. It was subsequently cooled to room temperature, and the volume was made up of 100 ml and centrifuged. We have pipetted out 0.2, 0.4, 0.6, 0.8, and 1 ml of the working standard into a series of test tubes, while we pipetted out 0.1 and 0.2 ml of the sample solution in two separate test tubes. Subsequently, the volume of each tube was 1 ml of water. A blank with 1 ml of water was prepared.

Moreover, 1 ml of phenol solution and 5 ml of 96% sulfuric acid were added to each tube and mixed thoroughly. The color intensity was measured at 490 nm. The total carbohydrate content in the sample was determined using a standard calibration curve.


$$\begin{array}{l} \text{The absorbance corresponds to }0.1\text{ ml of test }=\text{ X mg of glucose}. \end{array}$$

100 ml of the sample solution contains = X/0.1 × 100 mg of glucose = percent of the total carbohydrates present.

### 2.7 Estimation of ash

A sample of 1 g was weighed in the crucibles and burned in the muffle furnace between 500°C and 600°C to destroy all the organic material. We cooled the crucibles and took the weight. The weight difference would give the total ash content, which will be expressed as a percentage (18).

### 2.8 Hydration studies (cooking quality)

#### 2.8.1 Hydration capacity

Approximately 50 seeds were counted and transferred to a 200-ml conical flask and 100 ml of demineralized water. The flask was tightly stopped and left overnight (16 h) at room temperature. The next day, (i) the seeds were drained, (ii) the superfluous water was removed with the help of a paper towel, and (iii) the seeds were reweighed.


$$\begin{array}{l} \text{HC }=\;\left(\text{Weight after soaking }-\text{ Weight before soaking}\right)/50. \end{array}$$

Hydration capacity could be measured in terms of g water per seed.

#### 2.8.2 Hydration index

The hydration index is nothing but the ratio of the hydration capacity and the original weight of the seed.


HI=[(HC per seed/original weight per seed (g)].

#### 2.8.3 Swelling capacity

The swelling capacity is measured in terms of ml per seed. Approximately 50 seeds were counted and transferred to a 200-ml conical flask. Then, 100 ml of water was added to the conical flask.


$$\begin{array}{l} \text{SC}=\left(\text{Volume after soaking }-\text{ Volume before soaking}\right)/50. \end{array}$$

#### 2.8.4 Swelling index

The Swelling index is nothing but the ratio of the SC and volume.


SI=[(SC per seed/volume per seed (ml)].

#### 2.8.5 Cooking time (minutes)

For this study, 25 seeds were counted and soaked in 100 ml of demineralized water for 12 h. After 12 h, the samples were cooked at 100^0^C. The temperature was maintained constant throughout the process until the samples were cooked. The seeds were cooked until they became soft when pressed between the fingers to check for their softness.

### 2.9 Statistical analysis

The one-way analysis of variance (ANOVA) was carried out for each parameter to test the differences among the varieties and treatments. Using the Least Significant Difference (LSD) criteria, the superior variety and treatment were identified for each crop. Estimates of correlation were derived between different parameters to assess the depth of relationship among the parameters studied for each crop. Based on the estimates of correlation measured between different parameters, Principal Component Analysis (PCA) was carried out (19, 20) to explain the maximum variability in the data of parameters and identify the significant parameters of three legume crops, such as black gram, green gram, and pigeonpea. Statistical analysis was conducted using SYSTAT version 13 software.

## 3 Results and discussion

### 3.1 Effect of eCO_2_ and eT on ash, carbohydrates, crude fiber and protein content

Our study had a significant effect on protein, crude fiber, carbohydrate, and ash content due to eT + eCO_2_ and eT as compared to ambient (Ac). There was a negative impact of eT + eCO_2_ on protein and carbohydrate in three legume crops (black gram, green gram, and pigeon pea), while there was a significant positive impact of eT + eCO_2_ on crude fiber and ash content in three legume crops (Table 1). The magnitude of the negative effect on protein and carbohydrates is high in eT + eCO_2_ compared to eT. The protein content decreased by 25.6% under eT + eCO_2_, while the decrease was only 5.6% under eT compared to the control in blackgram. This clearly shows that the world's population may be at risk of protein deficiency because of eT + eCO_2_. A study revealed that specific inhibition of nitrate uptake and assimilation under eCO_2_ lowers N content in plant tissue (21). Moreover, Dong et al. (44) observed that under eCO_2_, there was a decrease in the protein content in vegetables to the extent of 9.5%. It is reported that eCO_2_ limits nitrogen uptake and the synthesis of nitrogenous compounds in vegetables and other crops (22, 45). This might be one of the reasons for a decrease in protein content under eT + eCO_2_ and eT in our study. A few studies have reported that under low N availability and eCO_2_, the extent of protein content reduction is higher than that of high N availability and eCO_2_. A meta-analysis study reported that protein concentration decreased with eCO_2_ in shoots and grain crops (46). Furthermore, it was reported that the concomitant increase in CO_2_ and temperature is likely to decrease the nutritional quality by decreasing shoot content and grain protein concentration due to poor root N-uptake rate. However, this decrease may be less in legumes than in cereals, as they can exchange C for N through N-fixing symbionts. The effect of rising atmospheric CO_2_ concentration on seed production and the fatty acid profiles of mung bean (Vigna mungo L. Wilczek) were studied by Ziska et al. (23). In Australia, eCO_2_ decreased Fe, Zn, P, and S concentrations in lentil and faba beans (47), and the degree of decrease was high under dry (20%−25%) compared to wet conditions. In alfalfa, the decrease in rubisco activity was associated with carbohydrate accumulation and depleted nitrogen availability (48). Increasing temperatures not only inhibit photosynthetic rate but enhance respiration rate under ambient CO_2_ conditions, causing the decline in the availability of carbohydrates for energy supply as well as carbon skeletons to support plant growth (49–51).

**Table 1:** Impact of eT and eCO_2_ on ash, carbohydrates, crude fiber, and protein content in black gram, green gram, and pigeon pea.

**Genoty pe**	**Treatment t**	**Ash (%)**	**Carbohydrates e (%)**	**Crude fiber**	**Protein**
**Black gram**					
LBG - 752	Control	2.33 ± 0.00	39.80 ± 7.56	1.06 ± 0.23	25.56 ± 0.96
	eT	2.55 ± 0.38	35.60 ± 3.40	2.73 ± 1.62	24.14 ± 0.99
	eT+eCO2	3.22 ± 0.69	37.60 ± 1.47	3.80 ± 0.72	19.02 ± 0.98
CD (p 0.05)		1.07	11.36	2.42	2.28
T - 9	Control	2.55 ± 0.38	37.60 ± 1.86	1.13 ± 0.50	24.62 ± 0.85
	eT	3.11 ± 0.19	33.03 ± 1.10	2.80 ± 0.69	22.20 ± 2.46
	eT+eCO2	3.55 ± 0.19	36.56 ± 0.57	2.06 ± 0.70	18.22 ± 1.71
CD (p 0.05)		0.63	3.02	1.49	4.21
**Green gram**					
LGG - 460	Control	2.44 ± 0.38	46.33 ± 2.51	1.20 ± 0.72	23.65 ± 0.32
	eT	3.44 ± 0.19	40.66 ± 9.71	1.93 ± 0.90	21.63 ± 0.81
	eT+eCO2	4.00 ± 0.00	45.66 ± 1.15	2.73 ± 1.10	19.48 ± 1.38
CD (p 0.05)		0.57	13.62	2.15	2.21
WGG - 42	Control	2.22 ± 0.19	42.16 ± 3.54	1.33 ± 0.57	22.73 ± 0.70
	eT	3.11 ± 0.84	39.00 ± 2.64	1.46 ± 0.23	19.93 ± 2.30
	eT+eCO2	3.73 ± 0.23	44.03 ± 1.90	2.73 ± 0.80	18.96 ± 2.12
CD (p 0.05)		1.20	6.50	1.37	4.23
**Pigeon pea**					
PRG - 176	Control	3.10 ± 0.78	44.25 ± 3.57	3.13 ± 0.83	23.37 ± 2.38

Carbohydrates are the major nutrients that support pollen development (52). The increase in photosynthesis caused by eCO_2_ results in increased carbohydrate production, which alters the plant's carbon and nitrogen metabolism (53). Seo et al. (54) reported that the elevated CO_2_ concentration could not compensate for the negative effect of elevated temperature and lowered the crude protein, crude fiber, and ash contents in radish. Elevated temperature or a combination of elevated temperature and elevated CO_2_ resulted in decreased seed protein and linolenic acid concentrations and increased oil and oleic acid in soybean cultivars. High temperatures may offset the positive effects of elevated CO_2_. In black gram, under eT, the carbohydrate and protein content were affected, while eT+eCO_2_ favored the ash content and fiber content. The increase in ash content was high to the extent of 38.19% under eT+eCO_2_ compared to eT, where the increase is up to 9.4%.

In contrast, the decrease in carbohydrate and protein content was 5.53% and 25.59% under eT + eCO_2_ compared to eT. In LBG-752, the decrease is up to 10.56% and 5.56% in carbohydrate and protein content. In our study, two black gram genotypes (LBG-752 and T-9) were studied, and we found that both were at par in carbohydrate, protein, crude fiber, and ash content in the control. Climate change positively affects grain legumes. Being C3 crops, they can allocate above-ground photosynthates to below-ground parts, such as roots and nodules, leading to improved plant biomass rhizospheric activities. Yet, the impact of eT on grain legumes is not consistently favorable. High temperatures, especially during the reproductive phase, negatively affect grain legume quality and performance (15).

Ash content increased by 9.44% and 21.96% in LBG-752 and T-9, respectively, under eT conditions, while the increase in ash content was to the extent of 38.19% and 39.21% in LBG-752 and T-9 genotypes of black gram under eT+eCO_2_ conditions. Under eT conditions, ash content increased by 40.9% and 40.1% in LGG-460 and WGG-42 genotypes of green gram, while the increase in ash content was to the extent of 63.9% and 68% in LGG-460 and WGG-42 genotypes of green gram under eT+eCO_2_ conditions. In the case of pigeon pea (PRG-176), ash content increased by 11.6% under eT and 13.9% under eT+eCO_2_ conditions compared to the control. Among the different genotypes studied, the ash content followed the order: WGG-42 > LGG-460 > T-9> LBG-752> PRG-176 under eT + eCO_2_ conditions. It is reported that key quality parameters in crops include the concentrations of crude fiber, protein, non-structural carbohydrates, and minerals. Pigeonpea grain is rich in carbohydrates, minerals, and proteins (55), with about 20%−26% protein, 65% carbohydrates, and 2% fats (56, 57). Thus, it is one of the best supplements to cereal-based diets (58). However, the decrease in carbohydrate content is observed to be significantly greater under eT as compared to eT+eCO_2_ conditions in both the genotypes of black gram and green gram and pigeon pea. In contrast, the extent of decrease in protein content is significantly greater under eT+eCO_2_ compared to eT conditions in all the genotypes studied.

Among the three legume crops studied under control, the carbohydrate content (46.33 g) was found to be higher in green gram than pigeon peas and black gram. The crude fiber (3.13) and ash content (3.10) were found to be higher in pigeon peas than green and black grams. The protein content was higher in black gram (25.56) than in pigeon peas and green gram. In green gram genotypes, significantly higher mean ash content (4.0 g) was attained by LGG−460 under eT+eCO_2_ compared to lower mean ash content (2.22 g) in WGG−42 under control. In pigeon peas, PRG−176 genotypes attained higher ash content (3.53 g) under eT+eCO_2_, while lower ash content (3.1 g) was observed under control. In black gram, the mean crude fiber content of genotype LBG−752 was significantly lower (1.06 g) under control, whereas a higher mean crude fiber content (3.8 g) was observed under eT+eCO_2_. In green gram, LGG−460 attained significantly lower mean crude fiber content (1.2 g) under control than higher mean content (2.73 g) under both LGG−460 and WGG−42 genotypes under eT+eCO_2_. In pigeon pea, PRG−176 genotypes had significantly lower mean crude fiber content (3.13 g) under control, whereas a higher mean fiber content (6.06 g) was observed under eT+eCO_2._

### 3.2 Effect of eCO_2_ and eT on phosphorus, calcium, and magnesium content

In our study, among the three legume crops studied, it was found that the green gram could maintain a higher P content that ranged from 325.5 to 406.6 mg/100 g across the treatments and genotypes and a Mg content that varied from 122.9 to 145.1 mg/100 g across the treatments and genotypes as compared to black gram and pigeon pea. However, the Ca content was found to be higher in black gram, ranging from 136.24 to 161.34 mg/100 g across the treatments and genotypes (Table 2). Mashifane et al. (59) reported that the grain of improved pigeon pea genotypes contained calcium (Ca) of 130 mg/100 g in the study. While the phosphorus, magnesium, and calcium content ranged from 2,716.66–4,473.49 mg/kg, 1,506.51–1,713.93 mg/kg, and 166.38–340.62 mg/kg in green gram varieties, as reported by nikarthil Sudhakaran et al. (60). The difference in P, Mg, and Ca content from one study to another might be attributed to variety, location, environmental conditions, soil type, and so on. The eT + eCO_2_ has resulted in a significant decrease in P, Ca, and Mg across the genotypes in our study. The degree of decrease is high compared to eT (Figures 1, 3). An experiment was carried out under free-air CO_2_ enrichment (FACE) conditions to study its impact on nutritional quality in soybean genotypes, and it was found that the eCO_2_ has decreased Ca, P, K, and Mg by 22.9, 9.0, 4.9, and 10.1% under eCO_2_ conditions, respectively (24). It is well said that eCO_2_, along with rising temperatures, has a huge impact on ecosystems, agriculture, and human health (25). Temperature increases lead to heat stress that can affect crop production and quality. If the heat stress coincides with critical growth stages, there would be a huge loss in terms of grain yield. It is reported that heat stress resulted in a 20% yield loss in wheat during the anthesis and grain-filling stage (26, 27). The combined effect of eT and eCO_2_ has resulted in a decline in P, Ca, and Mg content by 13.02%, 12.55, and 18.56%, respectively, in the LBG-752 genotype and by 13.1%, 14.38%, and 21%, respectively, in the T-9 genotype of black gram. In green gram, P, Ca, and Mg content were reduced by 17.74%, 25.47%, and 14.44%, respectively, in the LGG-460 genotype and by 18.53%, 35.88%, and 13.38%, respectively, in the WGG-42 genotype. In contrast, the reduction in terms of P, Ca, and Mg content is 6.63%, 15.0%, and 8.96%, respectively, in the PRG-176 genotype. The reduction was less in pigeonpea than in black and green gram. Altering the mineral content can significantly impact human and animal health. The decrease in the nutrient content in grain might be due to the dilution effect caused by eCO_2_ conditions and heat stress caused by eT.

**Table 2:** Effect of eT and eCO_2_ on hydration parameters in black gram, green gram, and pigeonpea.

**Genotype**	**Treatments**	**Hydration capacity (g water/seed)**	**Hydration index (g)**	**Swelling capacity (ml/seed)**	**Swelling index (ml)**	**Cooking time (min)**
**Black gram**						
LBG−752	Control	0.057 ± 0.000	0.98 ± 0.22	0.14 ± 0.01	1.09 ± 0.18	1.30 ± 0.04
	eT	0.055 ± 0.001	0.99 ± 0.09	0.13 ± 0.02	1.05 ± 0.16	5.34 ± 0.14
	eT+eCO_2_	0.051 ± 0.004	0.91 ± 0.18	0.07 ± 0.01	1.07 ± 0.22	5.46 ± 0.10
T−9	Control	0.054 ± 0.003	1.26 ± 0.20	0.11 ± 0.02	1.00 ± 0.10	2.60 ± 0.52
	eT	0.051 ± 0.006	1.06 ± 0.30	0.08 ± 0.02	0.99 ± 0.03	5.23 ± 0.18
	eT+eCO_2_	0.048 ± 0.003	1.14 ± 0.05	0.06 ± 0.01	0.91 ± 0.14	6.32 ± 0.08

### 3.3 Effect of eCO_2_ and eT on micronutrient (Zn, Fe, Mn, and Cu) content

**Figure 2:**
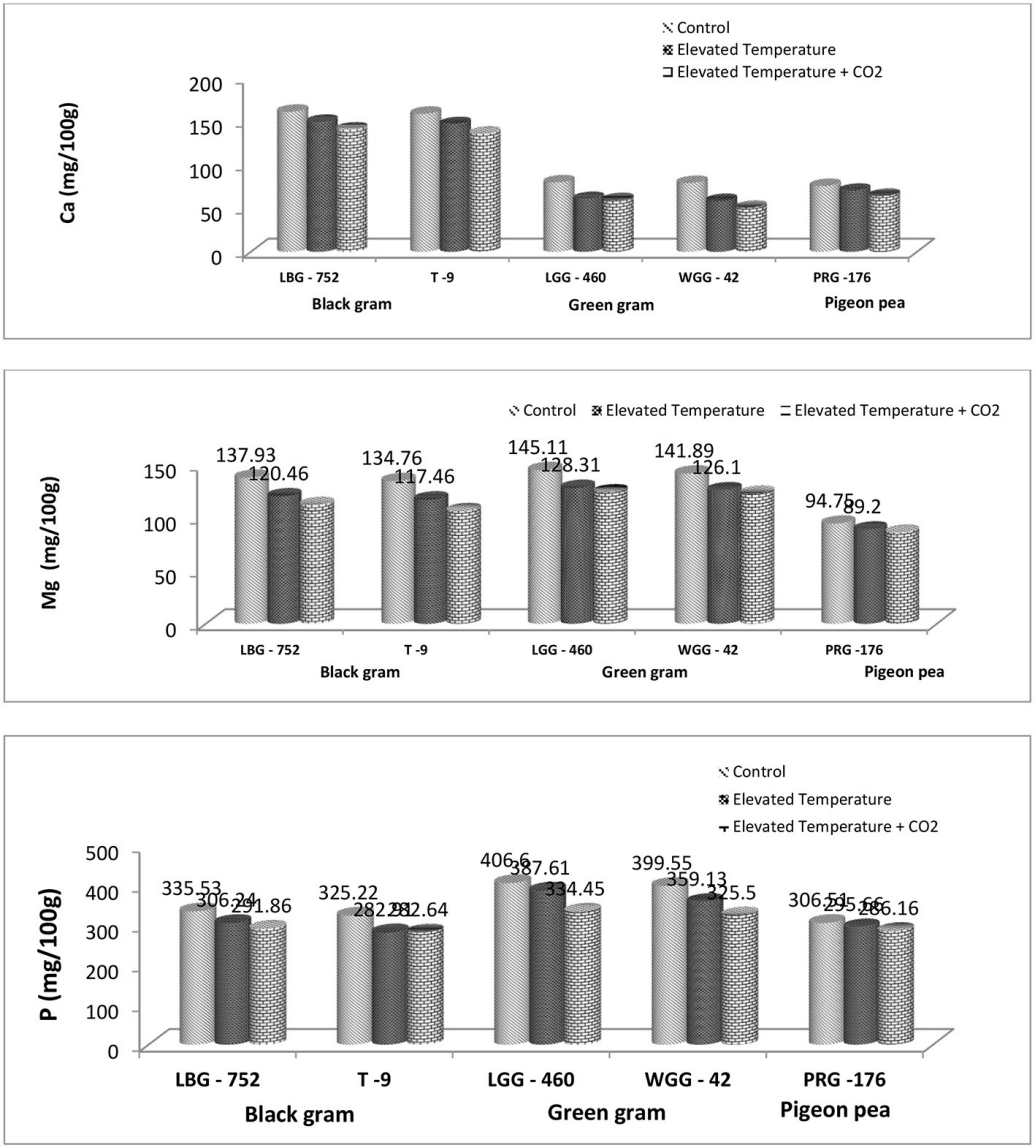
Effect of elevated CO_2_ and elevated temperature on mean calcium, magnesium, and phosphorus content (mg/100 g) on legume grains.

The eT and eT+eCO_2_ significantly negatively affected grain micronutrient content in all three crops, as shown in Figure 2. Among the genotypes studied, PRG-176 could maintain a higher Zn and Cu content. Moreover, the LBG-752 genotype of black gram maintained higher Fe content, and the LGG-460 genotype of green gram maintained higher Mn content under ambient conditions. This clearly shows the genotypic variability for micronutrient content (Figure 1). The proximate analysis in a study revealed iron and zinc content in mungbean genotypes ranged between 7.03–9.22 and 0.95–1.30 mg/100 g, respectively (28), while improved pigeon pea genotypes contained 5.23 mg/100 g iron (Fe) and 2.76 mg/100 g zinc (Zn), respectively (59). Another study revealed that Fe and Zn content in 26 black gram genotypes ranged from 71.02 to 100.20 ppm and 18.93 to 60.58 ppm, respectively (29). The combined effect of eT and eCO_2_ has resulted in a decline in Zn, Fe, Mn, and Cu content by 18.1%, 9.5%, 66.9%, and 50.53%, respectively, in the LBG-752 genotype and by 14.0%, 9.09%, 64.91%, and 27.71%, respectively, in the T-9 genotype of black gram. In green gram, Zn, Fe, Mn, and Cu content were reduced by 5.92%, 22.39%, 66.94%, and 60% in the LGG-460 genotype and by 14.9%, 19.7%, 70.09%, and 56.96%, respectively, in the WGG-42 genotype. In contrast, it was 15.77%, 17.45%, 21.05%, and 13.84%, respectively, in the PRG-176 genotype (Table 3). Overall, the reduction was less in pigeonpea than in black and green gram. The decrease in the nutrient content in grain might be due to the dilution effect caused by eCO_2_ conditions and heat stress caused by eT. It is reported that eCO_2_, or temperature, significantly affects the mineral accumulation and composition in rice grain (30–32). Under elevated temperature conditions, drought reduces the grain's N, P, Fe, and Zn content; hence, the total grain protein content decreases in maximum food legumes (33). Compared to Fe content in chickpea, black gram, and horse gram, which range from 5.97 to 8.76 mg/100 g, the Fe content is low in pigeon peas with 3.49 mg/100 g. However, the Zn content in pigeon peas is found to be 2.93 mg/100 g in the range of those pulses (34). However, the study states that under eCO_2_ + eT, seed Fe and Zn concentrations were restored to levels obtained under ambient CO_2_ and temperature conditions, suggesting that the potential threat to human nutrition by increasing CO_2_ concentration may not be realized (35). On the other hand, kernel oil (6.54% and 2.98%) and protein content (7.07% and 4.56%) declined at 550 and 700 ppm compared to ambient conditions in groundnut. Moreover, kernel iron and manganese content remained unchanged, while copper (13.93% and 26.19%) and calcium (24.33% and 8.20%) content declined at both elevated CO_2_ levels (36).

**Table 3:** Descriptive statistics of different parameters in black gram, green gram, and pigeonpea.

**Parameter**	**Minimum**	**Maximum**	**Mean**	**SD**	**CV (%)**
**Black gram**					
100 seed weight	102.5	117.6	109.4	3.4	3.1
Ash (%)	2.3	4.0	2.9	0.5	19.0
Carbohydrates	31.2	45.4	36.7	3.7	10.0
Crude fiber	0.6	4.6	2.3	1.2	54.3
Protein	16.3	26.6	22.3	3.1	14.0
Cooking time	1.3	6.4	4.4	1.9	42.3
Phosphorus (mg/100 g)	205.9	364.6	304.1	44.1	14.5
Iron (mg/100 g)	2.0	6.9	3.8	1.2	31.2
Zinc (mg/100 g)	1.9	4.6	2.9	0.8	26.7
Calcium (mg/100 g)	83.6	215.3	149.2	33.6	22.5
Magnesium (mg/100 g)	94.8	184.3	121.6	23.5	19.3
Manganese (mg/100 g)	0.3	1.8	0.8	0.4	52.2
Copper (mg/100 g)	0.3	1.0	0.7	0.2	32.9
**Green gram**					
100 seed weight	101.8	116.6	108.1	3.0	2.8
Ash (%)	2.0	4.0	3.2	0.7	23.7
Carbohydrates	30.0	49.0	43.0	4.7	10.9
Crude fiber	0.6	3.8	1.9	0.9	48.3
Protein	17.2	24.0	21.1	2.2	10.3
Cooking time	3.3	6.3	5.0	1.2	24.4
Phosphorus (mg/100 g)	258.0	450.8	368.8	50.8	13.8
Iron (mg/100 g)	2.1	3.8	2.7	0.4	14.2
Zinc (mg/100 g)	2.1	3.0	2.5	0.3	10.5
Calcium (mg/100 g)	45.6	122.4	65.1	19.4	29.7
Magnesium (mg/100 g)	94.7	159.1	131.4	18.7	14.3
Manganese (mg/100 g)	0.3	2.8	1.4	0.8	52.0
Copper (mg/100 g)	0.2	1.1	0.5	0.2	47.4
**Pigeon pea**					
Ash (%)	2.2	3.7	3.4	0.5	14.0
Carbohydrates	30.2	51.8	42.2	6.1	14.4
Crude fiber	2.2	6.8	5.0	1.6	32.7
Protein	18.9	24.9	20.9	2.3	10.8
Cooking time	6.4	13.4	10.1	2.7	27.0
Hydration capacity	0.07	0.13	0.10	0.02	15.3
Hydration index	0.64	1.64	1.12	0.34	30.8
Swelling capacity	0.18	0.22	0.19	0.01	7.7
Swelling index	0.89	1.28	1.03	0.12	12.0
Phosphorus (mg/100 g)	283.2	326.3	296.1	14.2	4.8
Iron (mg/100 g)	1.27	3.90	2.51	0.86	34.3
Zinc (mg/100 g)	2.07	3.89	3.29	0.52	16.0
Calcium (mg/100 g)	60.6	78.7	70.5	6.6	9.4
Magnesium (mg/100 g)	81.7	96.2	90.1	5.6	6.3
Manganese (mg/100 g)	0.60	1.67	0.99	0.31	30.9
Copper (mg/100 g)	0.96	1.39	1.19	0.14	12.0

### 3.4 Effect of eCO_2_ and eT on hydration parameters (cooking quality)

The impact of eCO_2_ and eT was studied on different hydration parameters (cooking quality) in black, green, and pigeon pea (Table 2).

#### 3.4.1 Hydration capacity

The hydration capacity was found to be significantly higher (0.0057 g water per seed) in LBG−752 of black gram under control, whereas lower hydration capacity (0.048 g water per seed) was found in T−9 under eT**+**eCO_2_ conditions. Green gram genotype LGG−460 was found to have a higher hydration capacity (0.04 g per seed) under control, whereas lower hydration capacity (0.03 g water per seed) was observed in WGG−42 under control, eT**+**eCO_2_, and eT conditions. The PRG−176 genotype of pigeon peas was found to have a higher hydration capacity (0.11 g water per seed) under control, whereas a lower hydration capacity (0.09 g water per seed) was observed under the elevated temperature regime.

#### 3.4.2 Hydration index

The hydration index (1.26 g) was found to be higher in T−9 of black gram under control, while it was lower (0.91 g) in LBG−752 under eT**+**eCO_2_. In green gram, LGG−460 had a significantly higher hydration index (1.43 g) under control, whereas it was lower (0.71 g) in WGG−42 under eT**+**eCO_2_ conditions. In pigeonpea, PRG−176 had a higher hydration index (1.33 g) under control and lower (0.8 g) under the eT situation.

#### 3.4.3 Swelling capacity

A swelling capacity of 0.14 ml per seed was observed in the LBG−752 genotype of black gram under control. It was significantly higher than a lower swelling capacity of 0.06 ml per seed observed in T−9 under eT**+**eCO_2_ conditions. Similarly, the two genotypes of green gram, such as LGG−460 and WGG−42, had a higher swelling capacity (0.15 ml per seed) under control compared to a lower swelling capacity (0.07 ml per seed) in WGG−42 under eT**+**eCO_2_ conditions. In pigeon peas, the PRG−176 genotypes had a higher swelling capacity (0.20 ml per seed) under control, which was significantly different, with a lower value of 0.18 ml per seed under eT**+**eCO_2_ conditions.

#### 3.4.4 Swelling index

The swelling index was found to be higher (1.09 ml) in the black gram genotype LBG−752 under control, while it was lower (0.91 ml) in T−9 under eT**+**eCO_2_ conditions. In green gram, the WGG−42 genotypes had a higher swelling index (1.96 ml) under control than a lower swelling index (1.03 ml) in the WGG−42 genotypes under eT**+**eCO_2_. A higher swelling index (1.07 ml) was observed in the PRG−176 genotypes of pigeon peas under control compared to a lower swelling index (0.98 ml) under eT**+**eCO_2_.

#### 3.4.5 Cooking time

The cooking time was found to be lower (1.3 min) in the LBG−752 genotype of black gram under control, whereas a higher cooking time (6.32 min) was observed for T−9 under the eT**+**eCO_2_. In green gram, a lower cooking time (3.35 min) was observed for LGG−460 under control, whereas a higher cooking time (6.26 min) was observed for WGG−42 genotype under eT**+**eCO_2_. Similarly, the PRG−176 genotype of pigeon peas had a significantly lower cooking time (6.84 min) under control than a higher cooking time (13.05 min) under eT+eCO_2_ conditions.

### 3.5 Distribution of different parameters in black gram, green gram, and pigeon pea

The descriptive statistics, such as mean, standard deviation, and coefficient of variation (%) of different parameters, were determined for black gram, green gram, and pigeonpea crops and are given in Table 3. In black gram, the 100 seed weight ranged from 102.5 to 117.6 with a mean of 109.4 (CV of 3.1%), while ash content ranged from 2.3 to 4.0 g with a mean of 2.9% (CV of 19.0%). The carbohydrates ranged from 31.2 to 45.4 g with a mean of 36.7 (CV of 10.0%), while crude fiber ranged from 0.6 to 4.6 g with a mean of 2.3 (CV of 54.3%). The protein ranged from 16.3 to 26.6 g with a mean of 22.3 (CV of 14.0%), while cooking time ranged from 1.3 to 6.4 min with a mean of 4.4 (CV of 42.3%). Among different nutrients, phosphorus ranged from 205.9 to 364.6 mg/100 g with a mean of 304.1 (CV of 14.5%), while iron ranged from 2.0 to 6.9 mg/100 g with a mean of 3.8 (CV of 31.2%); zinc ranged from 1.9 to 4.6 mg/100 g with a mean of 2.9 (CV of 26.7%); and calcium ranged from 83.6 to 215.3 with a mean of 149.2 mg/100 g (CV of 22.5%). Similarly, magnesium ranged from 94.8 to 184.3 mg/100 g with a mean of 121.6 (CV of 19.3%), while manganese ranged from 0.3 to 1.8 mg/100 g with a mean of 0.8 (CV of 52.2%), and copper ranged from 0.3 to 1.0 mg/100 g with a mean of 0.7 (CV of 32.9%).

In green gram, the 100 seed weight ranged from 101.8 to 116.6 g with a mean of 108.1 (CV of 2.8%), while ash content ranged from 2.0 to 4.0 g with a mean of 3.2 (CV of 23.7%). The carbohydrates ranged from 30 to 49 g with a mean of 43 (CV of 10.9%), while crude fiber ranged from 0.6 to 3.8 g with a mean of 1.9 (CV of 48.3%). The protein was in the range of 17.2 to 24.0 g with a mean of 21.1 (CV of 10.3%), while cooking time was in the range of 3.3 to 6.3 min with a mean of 5.0 (CV of 24.4%). Among different nutrients, phosphorus ranged from 258.0 to 450.8 mg/100 g with a mean of 368.8 (CV of 13.8%), while iron ranged from 2.1 to 3.8 mg/100 g with a mean of 2.7 (CV of 14.2%); zinc ranged from 2.1 to 3.0 mg/100 g with a mean of 2.5 (CV of 10.5%); and calcium ranged from 45.6 to 122.4 mg/100 g with a mean of 65.1 (CV of 29.7%). Magnesium was in the range of 94.7 to 159.1 mg/100 g with a mean of 131.4 (CV of 14.3%), while manganese was in the range of 0.3 to 2.8 mg/100 g with a mean of 1.4 (CV of 52.0%); and copper was in the range of 0.2 to 1.1 mg/100 g with a mean of 0.5 (CV of 47.4%).

In pigeon peas, ash content ranged from 2.2 to 3.7 with a mean of 3.4 (CV of 14.0%), while the carbohydrates ranged from 30.2 to 51.8 g with a mean of 42.2% (CV of 14.4%). The crude fiber ranged from 2.2 to 6.8 g with a mean of 5.0 (CV of 32.7%), while protein ranged from 18.9 to 24.9 with a mean of 20.9 (CV of 10.8%), and the cooking time ranged from 6.4 to 13.4 min with a mean of 10.1 (CV of 27.0%). The hydration capacity was in the range of 0.07 to 0.13 g water per seed with a mean of 0.10 (CV of 15.3%), while the hydration index was in the range of 0.64 to 1.64 g with a mean of 1.12 (CV of 30.8%). The swelling capacity ranged from 0.18 to 0.22 ml per seed with a mean of 0.19 (CV of 7.7%), while the swelling index ranged from 0.89 to 1.28 ml with a mean of 1.03 (CV of 12.0%).

Among different nutrients observed for pigeon pea, phosphorus ranged from 283.2 to 326.3 mg/100 g with a mean of 296.1 (CV of 4.8%), while iron ranged from 1.27 to 3.90 with a mean of 2.51 (CV of 34.3%). Zinc ranged from 2.07 to 3.89 mg/100 g with a mean of 3.29 (CV of 16.0%), while calcium ranged from 60.6 to 78.7 mg/100 g with a mean of 70.5 (CV of 9.4%). Magnesium was in the range of 81.7 to 96.1 mg/100 g with a mean of 90.1 (CV of 6.3%), while manganese was in the range of 0.60 to 1.67 mg/100 g with a mean of 0.99 (CV of 30.9%); and copper was in the range of 0.96 to 1.39 mg/100 g with a mean of 1.19 (CV of 12.0%).

### 3.6 Relationship between nutritional and cooking qualities

The relationship between different parameters in black gram, green gram, and pigeon pea has been derived and tested using a *t*-test. Only significant correlations observed between different parameters are given in Table 4. In black gram, positive and significant correlations were found to exist between (i) ash and cooking time (0.639^**^); (ii) crude fiber and cooking time (0.606^*^); (iii) protein and magnesium (0.500^*^); (iv) protein and manganese (0.681^**^); and (v) manganese and copper (0.545^*^). In green gram, positive and significant correlations were found to exist between (i) ash and crude fiber (0.606^*^); (ii) ash and cooking time (0.833^**^); (iii) crude fiber and cooking time (0.589^*^); (iv) protein and iron (0.593^*^); (v) protein and magnesium (0.608^*^); (vi) protein and manganese (0.760^**^); (vii) protein and copper (0.595^*^); (viii) phosphorus and manganese (0.497^*^); (ix) phosphorus and copper (0.577^*^); (x) iron and manganese (0.842^**^); (xi) iron and copper (0.528^*^); (xii) zinc and manganese (0.593^*^); (xiii) calcium and manganese (0.615^*^); and (xiv) manganese and copper (0.692^**^). In pigeon peas, positive and significant correlations were found to exist between (i) crude fiber and cooking time (0.781^*^), (ii) protein and swelling capacity (0.881^**^), and hydration capacity and hydration index (0.817^*^). Negative and significant correlations were found to exist between (i) ash content and phosphorus (−0.763^*^); (ii) crude fiber and protein (−0.816^*^); (iii) crude fiber and copper (−0.778^*^); (iv) crude fiber and swelling capacity (−0.883^**^); (v) cooking time and calcium (−0.787^*^); and (vi) cooking time and swelling capacity (−0.810^*^).

**Table 4:** Correlation between different parameters in black gram, green gram, and pigeonpea in Hyderabad.

**Parameter-1**	**Parameter-2**	**Black gram**	**Green gram**	**Pigeonpea**
Ash	Crude fiber		0.606^*^	
Ash	Cooking time	0.639^**^	0.833^**^	
Protein	Iron		0.593^*^	
Protein	Magnesium	0.500^*^	0.608^*^	
Protein	Manganese	0.681^**^	0.760^**^	
Protein	Copper		0.595^*^	
Protein	Swelling capacity			0.881^**^
Protein	Swelling index			
Phosphorus	Manganese		0.497^*^	
Phosphorus	Copper		0.577^*^	
Iron	Manganese		0.842^**^	
Iron	Copper		0.528^*^	
Zinc	Manganese		0.593^*^	
Calcium	Manganese		0.615^*^	
Manganese	Copper	0.545^*^	0.692^**^	
Hydration capacity	Hydration index			0.817^*^

Critical correlation value at the 5% level of significance with 16 degrees of freedom = 0.497. Critical correlation value at the 1% level of significance with 16 degrees of freedom = 0.623. Critical correlation value at the 5% level of significance with 7 degrees of freedom = 0.754. Critical correlation value at the 1% level of significance with 7 degrees of freedom = 0.875. ^*^ and ^**^ indicate significance at the 5% and 1% level of significance, respectively.

### 3.7 Principal components of different parameters in black gram, green gram, and pigeon pea

#### 3.7.1 Eigenvalues and variance explained by principal components

The principal components were determined as a function of different parameters and were given for black gram, green gram, and pigeon pea. Three principal components that were found to be significant and contributed to high variability were considered in this study (Figure 4). In accordance with PCA analysis, the interaction of PC1, PC2, and PC3 depicts the overall variance. In PCA analysis, different variables were studied: ash, carbohydrates, crude fiber, protein, cooking time, hydration capacity and swelling capacity, cooking time, hydration index, swelling index, phosphorus, iron, zinc, calcium, magnesium, manganese copper, and so on. The eigenvalues of principal components calibrated through different parameters ranged from 1.052 to 4.755 in black gram, while they ranged from 1.073 to 6.267 in green gram and ranged from 1.012 to 3.978 in pigeon pea. In PCA analysis, PC1 and PC2 explained 62.1% of the total variance, as shown in Figure 5. PC1 and PC3 showed 61.1% of the total variance, as shown in Figure 5, while PC2 and PC3 explained 50.8% of the total variance, as shown in Figure 5. The extent of the strong relationship is clear in Figure 5. If grouped, treatment and genotype of black gram, greengram, and redgram. The varieties of red gram, black gram, and green gram were plotted against three principal components (PC1, PC2, and PC3), revealing that red gram forms a distinct cluster, indicating it differs significantly from the other two regarding the variables contributing to these components. While black gram and green gram show some overlap in variability, they are more clearly separated along PC1. The dispersion of varieties within each cluster highlights significant variability due to the treatments. Black gram and green gram are relatively well-clustered in all plots, suggesting similar internal variability. However, the confidence ellipses show that green gram exhibits greater variance than black gram. The red gram displays the least variance, as reflected by the tighter clustering of its points. The maximum parameters were found to be positively loaded on the two leading principal components, PC1 and PC2, calibrated for the data of black gram, green gram, and pigeon pea in the study. The percentage of explained variance was 36.2% for PC1, 25.9% for PC2, and 24.9% for PC3. The major contributors to PC1 are Mg, crude fiber, cooking time, phosphorus, hydration capacity, Ash content, and Mn. The major contributors to PC2 are swelling capacity, Cu, Mn, carbohydrate, hydration capacity, and Zn, while the major contributors to PC3 are Ca, Fe, Zn, protein, carbohydrate, swelling index, and ash content (Figure 6). Moreover, the scree plot, where the line plot of the correlation matrix's eigenvalues is arranged from greatest to smallest, is shown in Figure 7.

**Figure 3:**
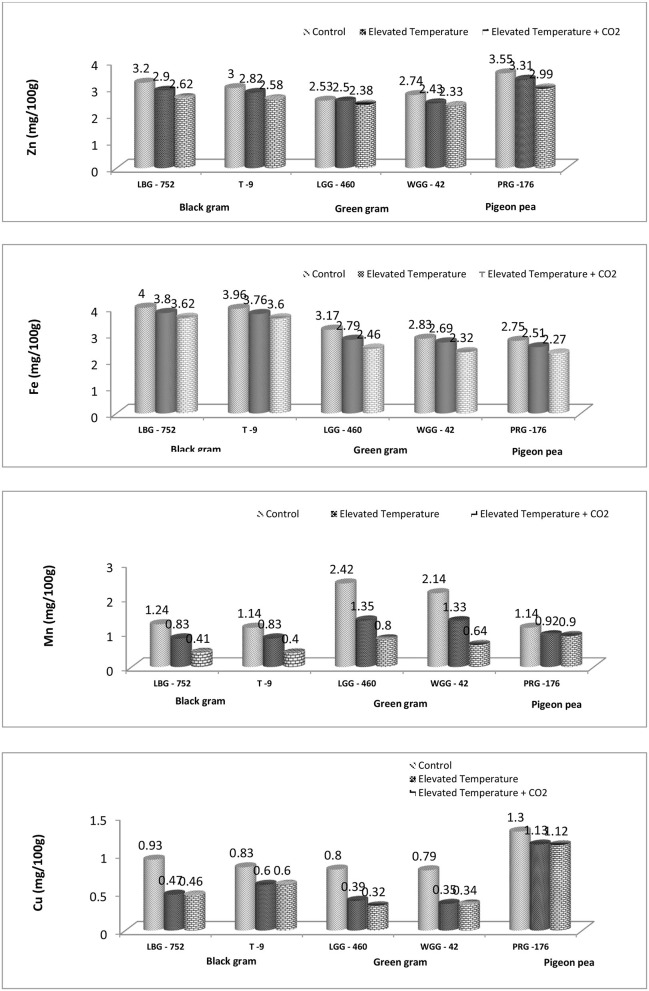
Effect of eCO_2_ and elevated temperature on mean Zn, Fe, Mn, and Cu content (mg/100 g) in legume grains.

**Figure 4:**
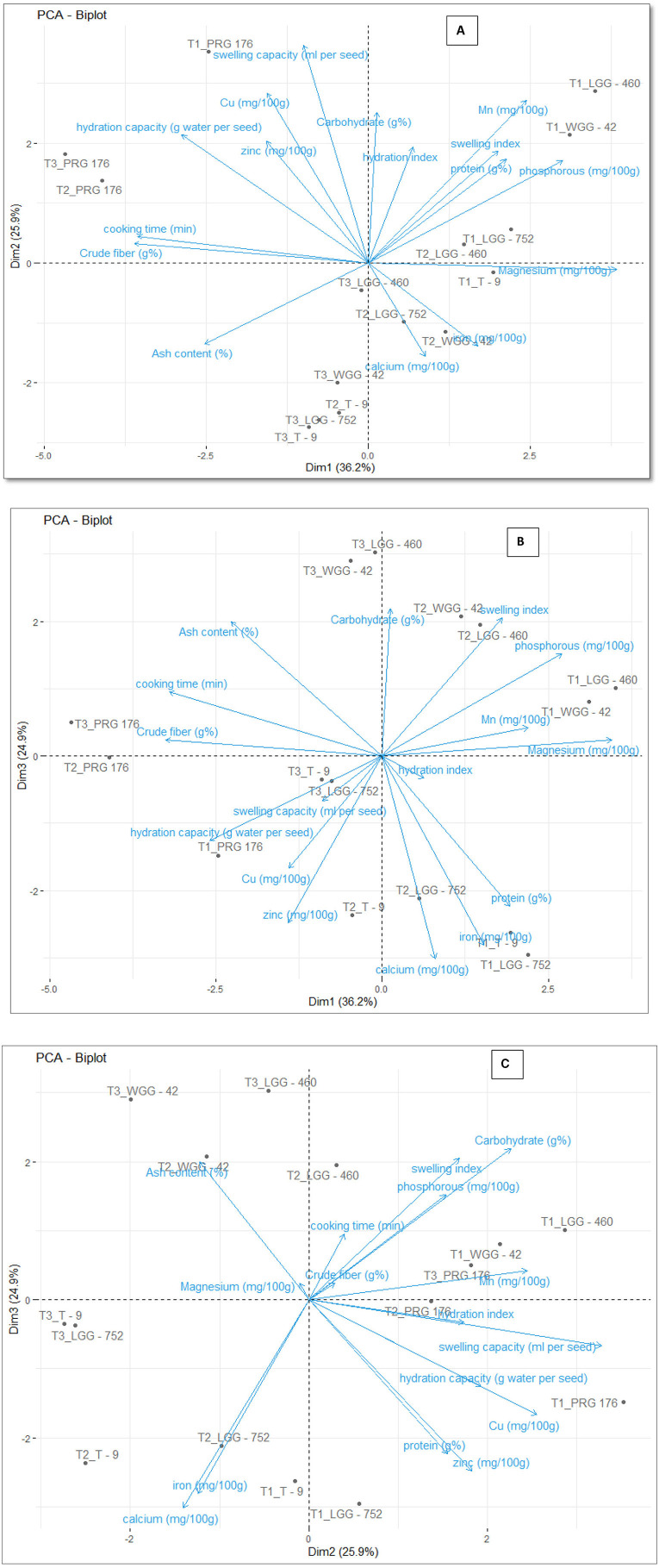
A group of three biplot panels displaying the PC loadings and component scores. **(A)** PC1 vs. PC2 explains the highest variability (i.e., 36.2% and 25.9%, respectively); **(B)** PC1 vs. PC3; and **(C)** PC2 vs. PC3 illustrates moderate variation (i.e., 25.9% and 24.9%, respectively). Highly correlating variables form a small angle between the rays and may often overlap, as seen in the right quadrant. Long rays indicate the magnitude of the specific variable loading.

**Figure 5:**
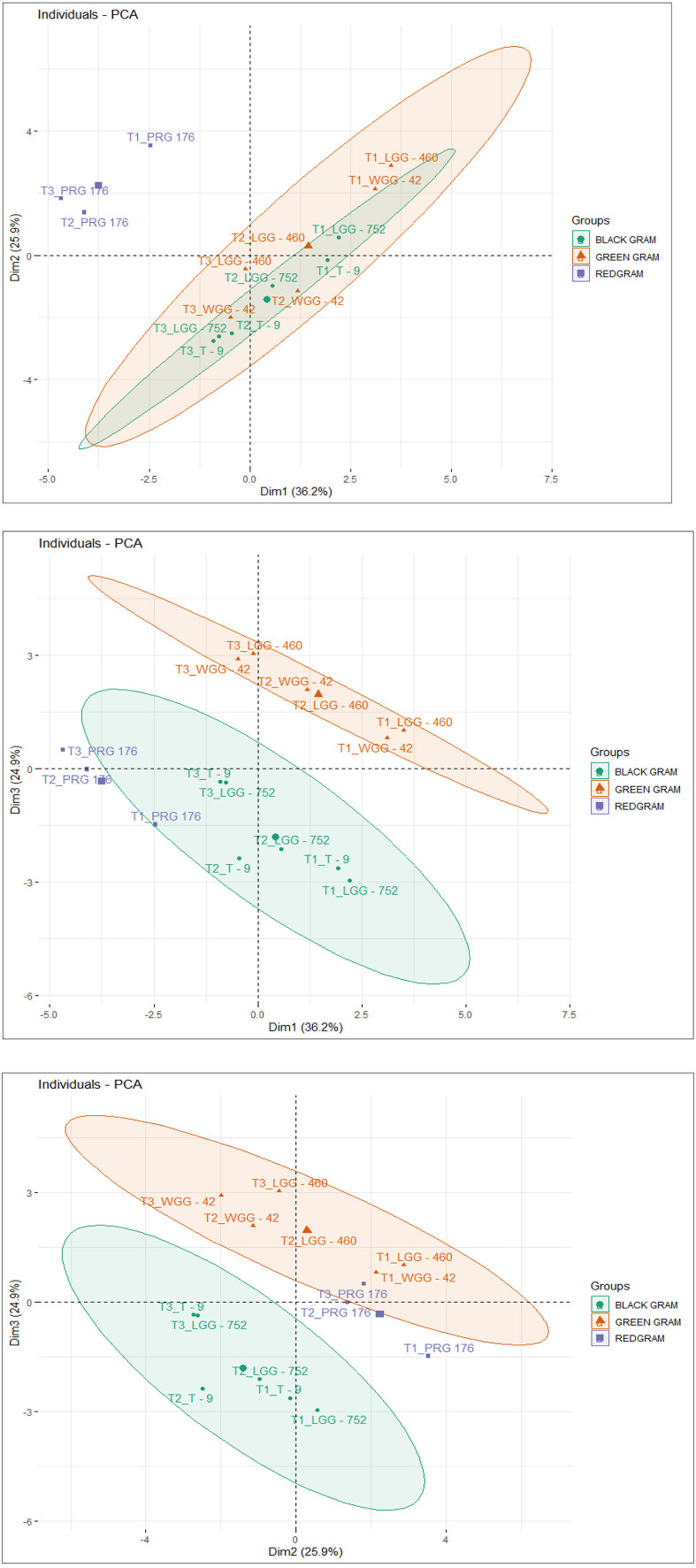
Cluster plot of black gram, green gram, and redgram.

**Figure 6:**
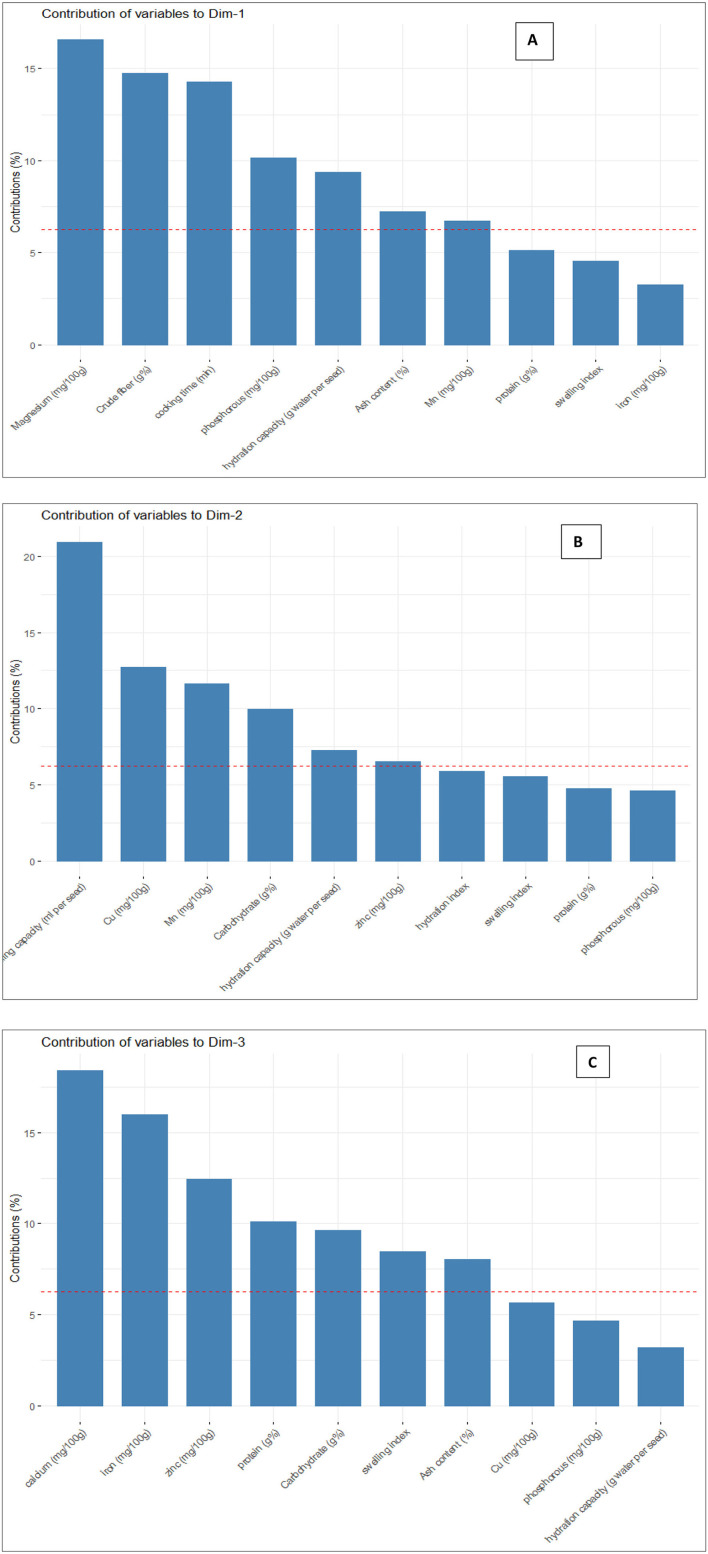
Contribution of different variables for **(A)** PC1, **(B)** PC2, and PC **(C)**.

**Figure 7:**
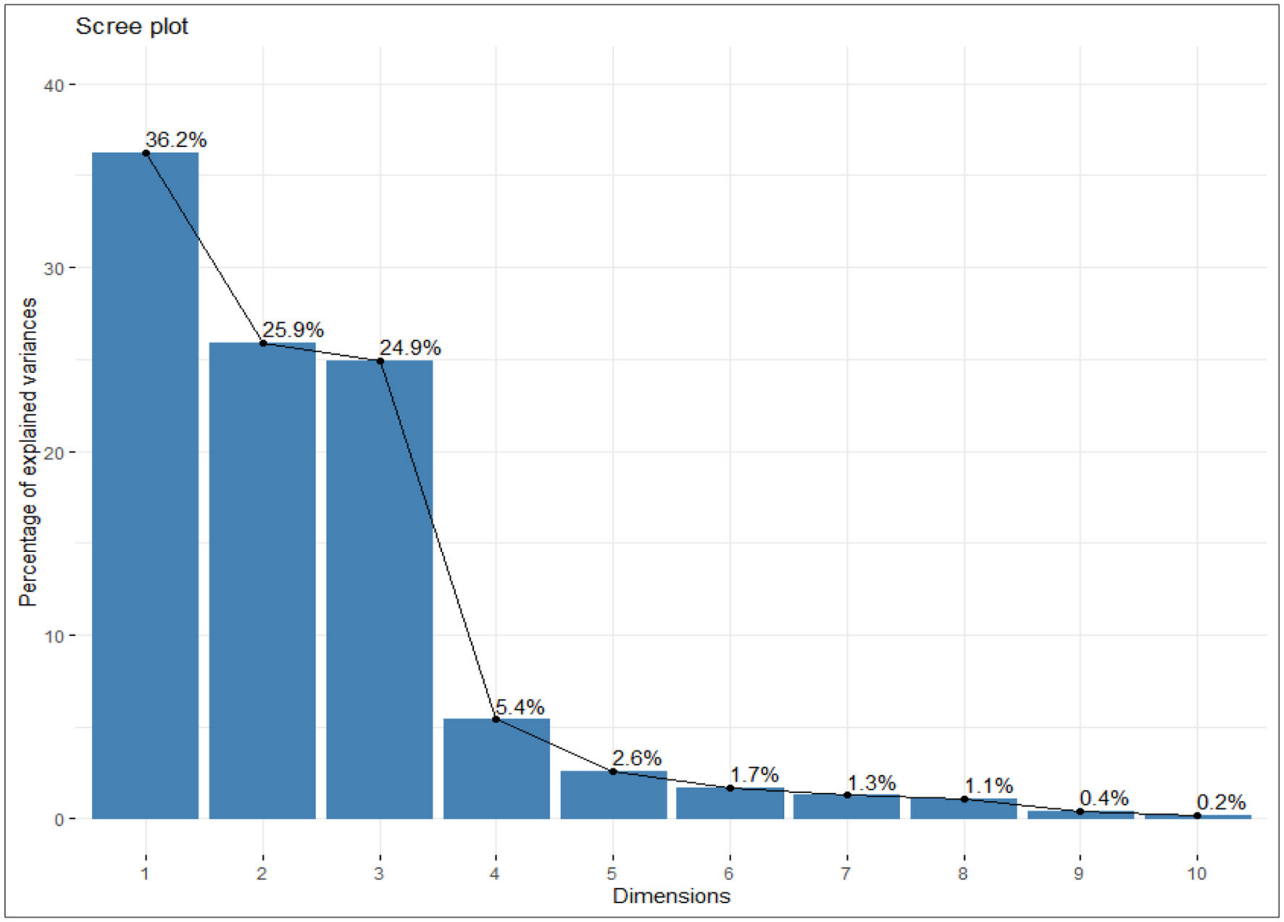
Scree plot showing the percentage of explained variability among the different parameters studied.

## 4 Conclusion

Climate change and global warming are projected to negatively impact humans as plants alter their chemical composition in response to elevated CO_2_ and high temperatures. In our study, eCO2 and eT levels were found to adversely affect the nutritional quality of legumes, highlighting the need for proper nutrient management and genetic improvements. The elevated conditions led to a significant decline in the concentrations of protein, phosphorus, and essential micronutrients, including iron, zinc, calcium, and copper. Additionally, the cooking quality of legumes was also negatively impacted by these conditions.

Among the three legumes studied, pigeon peas (PRG-176) demonstrated a greater ability to withstand elevated CO_2_ and temperature conditions. The findings suggest that under such conditions, semi-arid alfisols will require increased fertilizer application to counteract the negative effects on grain quality. This approach could help improve the mineral nutrient composition affected by nutrient dilution.

## Data Availability

The data analyzed in this study is subject to the following licenses/restrictions: will be provided on request. Requests to access these datasets should be directed to K. Sreedevi Shankar- sreedevikobaku@gmail.com.
