# Supplementary material for: Change in mineral composition and cooking quality in legumes grown on semi-arid alfisols due to elevated CO2 and temperature
Source: Front Nutr. 2025 Jan 6;11:1444962. doi: 10.3389/fnut.2024.1444962 (PMC11742948; doi:10.3389/fnut.2024.1444962)
Supplement: Supplementary file 1 [file Data_Sheet_1.docx]

**Supplementary table**

**Table: Treatment effect on nutrient content and cooking quality in the three legume crops**

| **Black Gram** | | | | | | | | | | | | | | | | |
| --- | --- | --- | --- | --- | --- | --- | --- | --- | --- | --- | --- | --- | --- | --- | --- | --- |
| **Trt** | **Ash content (%)** | **Carbohydrate (g%)** | **Crude fiber (g%)** | **protein (g%)** | **iron (mg/100g)** | **zinc (mg/100g)** | **calcium (mg/100g)** | **Magnesium (mg/100g)** | **Mn (mg/100g)** | **Cu (mg/100g)** | **phosphorous (mg/100g)** | **cooking time (min)** | **hydration capacity (g water per seed)** | **hydration index** | **swelling capacity (ml per seed)** | **swelling index** |
| T1 | a | a | a | a | a | a | a | a | a | a | a | b | a | a | a | a |
| T2 | a | a | a | a | b | ab | b | b | b | a | a | a | a | a | a | a |
| T3 | a | a | a | b | c | b | b | b | c | a | a | a | a | a | a | a |
| **Green Gram** | | | | | | | | | | | | | | | | |
| **Trt** | **Ash content (%)** | **Carbohydrate (g%)** | **Crude fiber (g%)** | **protein (g%)** | **iron (mg/100g)** | **zinc (mg/100g)** | **calcium (mg/100g)** | **Magnesium (mg/100g)** | **Mn (mg/100g)** | **Cu (mg/100g)** | **phosphorous (mg/100g)** | **cooking time (min)** | **hydration capacity (g water per seed)** | **hydration index** | **swelling capacity (ml per seed)** | **swelling index** |
| T1 | b | a | b | a | a | a | a | a | a | a | a | c | a | a | a | a |
| T2 | a | a | b | ab | ab | a | b | b | b | b | ab | b | a | a | a | a |
| T3 | a | a | a | a | b | a | b | b | c | b | b | a | a | a | a | a |
